# In-Line Detection of Bed Fluidity in Gas–Solid Fluidized Beds Using Near-Infrared Spectroscopy

**DOI:** 10.3390/pharmaceutics15092246

**Published:** 2023-08-30

**Authors:** Hao Fu, Kaixuan Teng, Jie Zhao, Sheng Zhang, Haibin Qu

**Affiliations:** 1 Pharmaceutical Informatics Institute, College of Pharmaceutical Sciences, Zhejiang University, Hangzhou 310058, China; 2 Innovation Institute for Artificial Intelligence in Medicine of Zhejiang University, Hangzhou 310018, China

**Keywords:** gas–solid fluidized bed, near-infrared diffuse reflectance spectroscopy, voidage, defluidization, fluidity, in-line monitoring

## Abstract

A novel approach was developed to detect bed fluidity in gas–solid fluidized beds using diffuse reflectance near-infrared (NIR) spectroscopy. Because the flow dynamics of gas and solid phases are closely associated with the fluidization state, the fluidization quality can be evaluated through hydrodynamic characterization. In this study, the baseline level of NIR spectra was used to quantify the voidage of the fluidized bed. Two indicators derived from the NIR baseline fluctuation profiles were investigated to characterize bed fluidity, named bubble proportion and skewness. To establish a robust fluidity evaluation method, the relationships between the indicators and bed fluidity were investigated under different conditions firstly, including static bed height and average particle size. Then, a generalized threshold was identified to distinguish poor and good bed fluidity, ensuring that the probability of the α- and β-errors was less than 15% regardless of material conditions. The results show that both indicators were sensitive to changes in bed fluidity under the investigated conditions. The indicator of skewness was qualified to detect bed fluidity under varied conditions with a robust threshold of 1.20. Furthermore, the developed NIR method was successfully applied to monitor bed fluidity and for early warning of defluidization in a laboratory-scale fluidized bed granulation process.

## 1. Introduction

Gas–solid fluidized beds are widely used in various industries, such as the chemical, pharmaceutical, and food industries, because of their excellent mass and heat transfer efficiency. The typical applications of gas–solid fluidized beds include polymerization, granulation, and drying of particles. Maintaining good fluidization quality is the basis of fluidized bed processes. However, in practice, changes in fluidized material properties or operating conditions may lead to a decrease in fluidization or even unscheduled disruptions of production. One of the reasons for fluidization degradation is the increase in interparticle forces. For instance, the incremental addition of liquid to a fluidized bed of Geldart group B particles can cause a transition to group A behavior and eventually to group C behavior [1]. Particle agglomeration is another common problem [2,3]. The increase in particle size leads to a higher minimum fluidization velocity that in turn degrades the fluidization. The deterioration of bed fluidity directly indicates a loss of fluidization. Therefore, a prompt adjustment of the operating conditions during fluidized bed processes is essential to maintain the bed fluidity and prevent costly shutdowns of the entire installation.

Increasing superficial gas velocity is an effective strategy to maintain fluidization quality. Sufficient superficial gas velocity promotes the fluidization of granules, which ensures that the drag force exerted on the particles is kept in a dynamic balance with the gravity, the interparticle force, and the force between the particle and bubble [4]. Boyce et al. [5] presented the validity of keeping the ratio of superficial gas velocity (*U*) and minimum fluidization velocity (*U*_mf_) constant across dry and wet fluidized beds to reproduce the same or comparable hydrodynamics. Generally, the superficial gas velocity is required to be at least three times greater than *U*_mf_ in most fluidized bed processes. Nevertheless, due to the dynamic changes in material properties, it is challenging to obtain the minimum fluidization velocity in real time during commercial production processes. Traditionally, superficial gas velocity was adjusted manually or routinely based on the operator’s experience, which is subjective and high-risk. Hydrodynamic characterization provides another approach for superficial gas velocity adjustment. Excellent bed fluidity means vigorous interphase momentum exchange related to flow dynamics, such as bubble evolution, particle movement, and dispersion. In contrast, inactive gas or solid phase motions indicate poor fluidization quality. Various kinds of signals related to the hydrodynamic properties of the gas or solid phase have been investigated for the early detection of defluidization, such as pressure [6,7,8,9,10], temperature [11], triboelectric current [12], acoustic emission [13], vibration [14,15,16], and electrical conductance [17,18]. In these studies, specific signal analysis methods were used to extract informative signal features, which varied with fluidized bed hydrodynamics. Then, the loss of fluidization was associated with abnormal signals, assisting operators in adjusting operating conditions, such as superficial gas velocity.

Bed voidage fluctuation is a common phenomenon dominated by bubble evolution in gas–solid fluidized beds. Wang et al. [19] studied the typical fluidization behaviors under different superficial gas velocities by the numerical method. The simulation results showed that a higher superficial gas velocity led to more intense fluctuations in the void fraction at different bed heights of the bubble-fluidized bed. Therefore, it is possible to determine whether the superficial gas velocity is sufficient to obtain a steady fluidization state by analyzing the fluctuation of bed voidage. In general, researchers apply fiber optic measures for the gas-phase characteristics in gas–solid fluidized beds. Fiber optical probes have been successfully applied to record fluctuations in bed voidage. Furthermore, the hydrodynamic parameters of the bubbles could be extracted from the voidage signals, such as bubble frequency, bubble chord length, and bubble rise velocity [20]. More detailed descriptions of the basics and applications of fiber optical sensors can be found in the review paper by Golshan et al. [21]. However, the main drawback of fiber optic measurements is that the intrusive probe may be contaminated by cohesive materials or damaged by high-density particles.

As another widely used optical method, near-infrared (NIR) spectroscopy, has been proved to be an efficient process analytical technology (PAT) tool in the petrochemical, food, and pharmaceutical industries [22,23,24]. Diffuse reflectance NIR spectroscopy is a completely noninvasive NIR analysis technique that is commonly applied for analyzing static granules and flowing powders. In diffuse reflection mode, the light source and detector are located on the same side of the sample. The radiation interacts with the particles and may be absorbed or transmitted or may undergo diffuse reflection. As a result, spectral bands are observed due to the absorption of radiation, while transmission and diffuse reflection are determined by the physical characteristics of the sample, including the void fraction [25], which causes spectral baseline drifting. Thus, both chemical and physical information can be extracted from diffuse reflectance NIR spectra. Based on diffuse reflectance NIR spectroscopy, in-line analysis methods have been developed for moisture content [26,27], active pharmaceutical ingredient [28], particle size distribution [29,30], and bulk density [31] quantification during fluidized bed processes. In addition, several studies have been published on monitoring powder-handling processes using diffuse reflectance NIR spectroscopy. Ropero et al. [32] used diffuse reflectance NIR spectroscopy to develop an in-line characterization method of powder-flow behavior. The flow interruptions in the powder-voiding process were measured based on observations of large changes in the baseline and increases in the noise for powders that flow poorly. Inspired by the above-mentioned literature, diffuse reflectance NIR spectroscopy can potentially be used to characterize bed voidage fluctuations based on the relationship between the baseline level and interparticle voids. However, to the best of our knowledge, there have been no reports of the use of diffuse reflectance NIR spectroscopy to detect bed fluidity in gas–solid fluidized beds.

In this study, a novel approach based on diffuse reflectance NIR spectroscopy was established for fluidity characterization in a gas–solid fluidized bed. The NIR spectra of fluidized material were used to distinguish poor and good bed fluidity. Two fluidity indicators were derived from the baseline fluctuation profiles of NIR spectra, based on the marked differences in bed voidage between various fluidities. Furthermore, an in-line fluidity evaluation method was established for early warning of defluidization. Thus, the NIR spectra used for quantifying granule attributes can also provide bed fluidity information in fluidized bed applications.

## 2. Materials and Methods

### 2.1. Materials

In this study, polypropylene (PP) particles (Sinopec Maoming Petrochemical Company, Maoming, China) with a true density of 910 kg/m^3^ and an average particle size (*d*_p_) specification of 630, 805, and 1040 μm were used as fluidized materials.

### 2.2. Experimental Setup

The experiments were performed using a laboratory-scale fluid-bed granulator (FBLZ-3, Chanse Technology Co., Ltd., Changzhou, China). The chamber was made of stainless steel and consisted of a wind box, a conical chamber, and an expansion chamber. The conical chamber had a cone apex of 18.8° with a 14.0 cm inlet diameter and a 32.5 cm outlet diameter. A sight window made of quartz was embedded in the wall of the conical chamber for visual observations.

**Figure 1:**
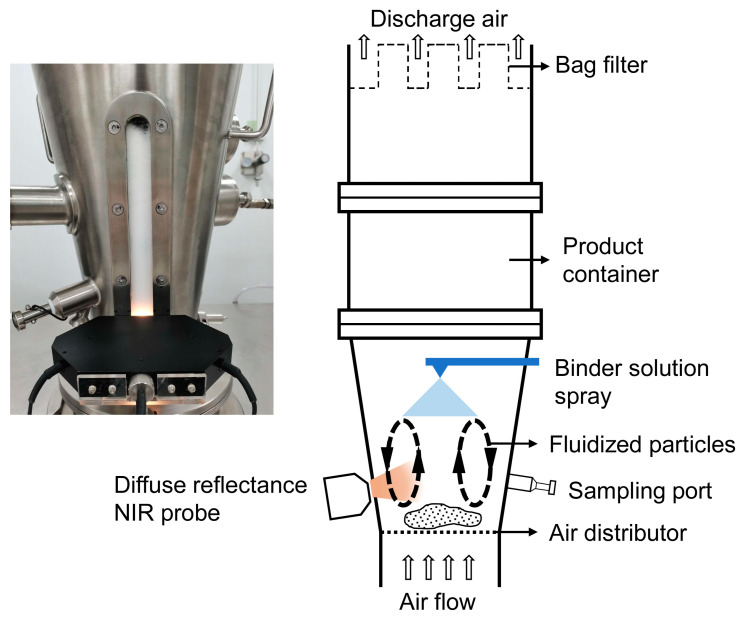
Laboratory-scale fluid-bed granulator equipped with diffuse reflectance NIR spectroscopy.

The diffuse reflectance NIR spectra were measured by a customized NIR probe (Beijing XingYuan AoTe Technology Co., Ltd., Beijing, China) mounted flush to the sight window 11 cm above the air distributor, as shown in Figure 1. The probe consisted of a receiving optical fiber installed in the direction of the symmetry axis and two tungsten halogen light sources symmetrically positioned at a 45° angle from the optical fiber. The probe was customized to make the incident NIR light focus on the inner surface of the sight window. A grating detector (AvaSpec-NIR256-1.7-EVO, Avantes, Apeldoorn, The Netherlands) was used for NIR light intensity detection. The diffuse reflectance NIR spectra were recorded as log_10_(1/*R*), where *R* is the reflectance over the wavelength range of 895.2 nm to 1854.2 nm (251 data points). The spectral region selected in the study has larger effective sample size compared to visible and short-wave NIR light. This is because less light will be scattered at longer wavelengths, yielding deeper penetration depth. The detector was controlled via LabVIEW (National Instruments, Austin, TX, USA). Spectra were collected by averaging 16 scans of 0.003 s integration time at a sample rate of 3.25 Hz. Reference spectra were obtained with a Teflon plate before each experiment. Spectral data ranging from 895.2 nm to 1680.8 nm (consisting of 203 wavelength points) were selected for further analysis according to the signal-to-noise ratio.

### 2.3. Experimental Procedure

In this study, two series of experiments were conducted: calibration experiments and validation experiments. In calibration experiments, the NIR spectra of PP particles collected under different fluidization conditions were used to establish a robust fluidity evaluation method. In each experiment, after determining the minimum fluidization velocity via the pressure drop method, the superficial gas velocity was set from 1.5 to 6.0 *U*_mf_. At each gas velocity, a total of 1560 diffuse reflectance NIR spectra were collected for 8 min with a sampling rate of 3.25 Hz. A summary of the experimental conditions is provided in Table 1. Visual observations of the bed hydrodynamics were performed to characterize the bed fluidity at each *U*, which are described in detail in Section 3.1. Specifically, the sensitivity of bed fluidity with respect to changes in average particle size (*d*_p_) and static bed height (*H*_b_) was also investigated.

For validation experiments, five experiments were performed to evaluate the robustness of the fluidity detection method with respect to particle size and static bed height. PP particles were selected as fluidized materials. The conditions of each experiment are listed in Table 2. The superficial gas velocity of each experiment was initialized as 1.5 *U*_mf_. Once the bed had been fluidized for 10 min, the superficial gas velocity was increased by 0.5 *U*_mf_. The gas velocity was increased stepwise until 6.0 *U*_mf_. Thus, each validation experiment lasted for 100 min, and the bed fluidity gradually improved from poor to good. In addition, to demonstrate the applicability, the NIR method was also applied in two laboratory-scale fluidized bed granulation processes: a gas-velocity-increasing granulation process with excellent fluidization quality and a bed-fluidity-decreasing granulation process with constant gas velocity, ending with bed collapse. The NIR spectra were collected at the spraying stage, during which the particle moisture, average particle size, and static bed height gradually increased.

### 2.4. Signal Processing

**Figure 2:**
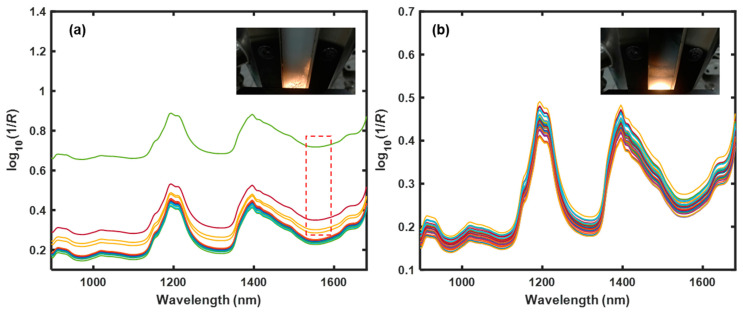
(**a**) Near-infrared (NIR) spectra of the fluidized process with good fluidity. Due to the occurrence of bubble events, there are several spectra with high baseline levels (red dashed box). (**b**) NIR spectra of the fluidized process with poor fluidity. Because the emulsion phase occupies the irradiated area, all spectra show low baseline levels. The color lines refer to NIR spectra collected in a certain interval.

Complex interactions occur between the gas and solid phases in the fluidized bed. The diffuse reflectance NIR spectra are affected by the sample morphology near the irradiated area. Baseline shifting is the main observed phenomenon; it results from transmission and diffuse reflection effects and can reflect variations in the voidage. Figure 2a shows the NIR spectra obtained from a fluidized process with good fluidity. There were several NIR spectra with high baselines. Once a bubble partially or completely occupied the irradiated area, the void fraction approached 1, and the spectral baseline evidently shifted due to the transmission effect. Figure 2b shows the NIR spectra obtained from a fluidized process with poor fluidity, where bubbles rarely appeared at the probe position near the wall and the emulsion phase was always illuminated. As a result, the voidage fluctuated in a certain range due to the dynamic characteristics of the fluidized particles. All NIR spectra showed low baseline levels. Thus, the baseline levels of diffuse reflectance NIR spectra could be used to characterize the fluidity of the gas–solid fluidized bed by indicating the change in voidage.

**Figure 3:**
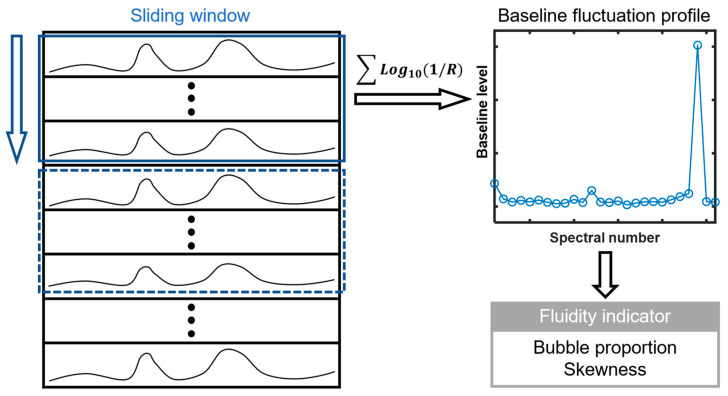
Schematic diagram of signal processing. The blue solid and dashed boxes refer to two adjacent sliding windows.

For in-line application, a sliding window was used to divide the continuously acquired NIR spectra into subsets. The width of the sliding window was set to 26, corresponding to a time interval of 8 s. The window swapped 26 spectra at each step. The baseline level (*bl*) of the NIR spectra was quantified as the sum of log_10_(1/*R*) values at all 203 spectral wavelengths. Then, two fluidity indicators were derived from the baseline fluctuation profiles of each sliding window. A schematic diagram of signal processing is shown in Figure 3. The calculation methods of each indicator are described in detail below.

#### 2.4.1. Bubble Proportion

The intensity of the bubble movement relates to the bed fluidity. Once the bed fluidity deteriorates, bubbles tend to pass through the center of the bed or channels. Thus, the risk of decrease in fluidization is negatively correlated with the frequency of bubble events near the wall. In this study, the bubble behavior intensity was quantified by an indicator called the bubble proportion, which was defined as the ratio of the number of times the bubble phase was observed at the probe position to the total number of spectra within the sliding window, as follows:

(1) $$P_{b}=m_{b}/m_{w}*100\%$$

where *P_b_* is the bubble proportion, *m_b_* is the number of spectra in bubble events, and *m_w_* is the number of spectra in a sliding window.

**Figure 4:**
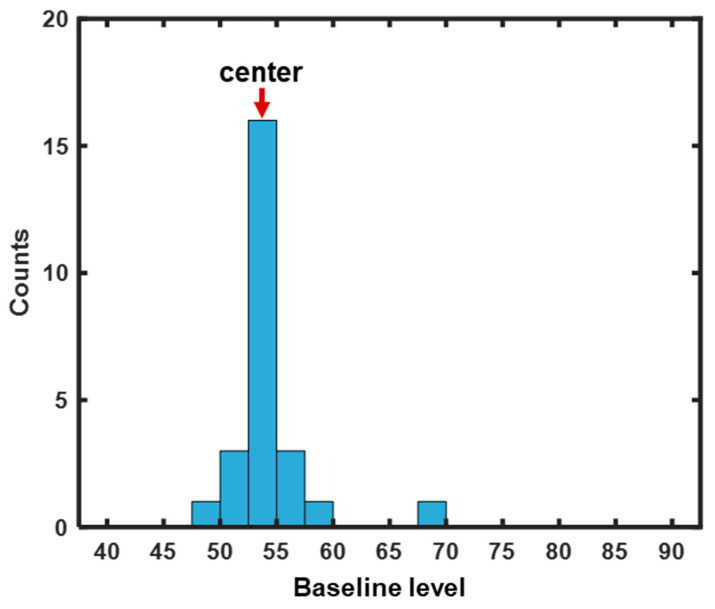
Histogram of the baseline level within a sliding window.

To detect the bubble phase, a bubble event detection algorithm [33] was used to extract information on the beginning and end of a bubble event from the baseline fluctuation profile. The steps of the bubble detection method are described in detail as follows:

The first task was to identify the central location of the baseline level. However, the baseline level was affected not only by bubble events but also by changes in the physicochemical attributes of the bed material. Thus, to obtain the position of the center, a histogram plot was generated for the baseline level within each sliding window, as shown in Figure 4. The horizontal coordinate ranged from 40 to 90, including positions where the center was located. The histogram consisted of 20 single bars that were equally distributed over the range of the filtered signal. The center was then identified as the average of the left and right edges of the bin with the most frequently occurring baseline level.

**Figure 5:**
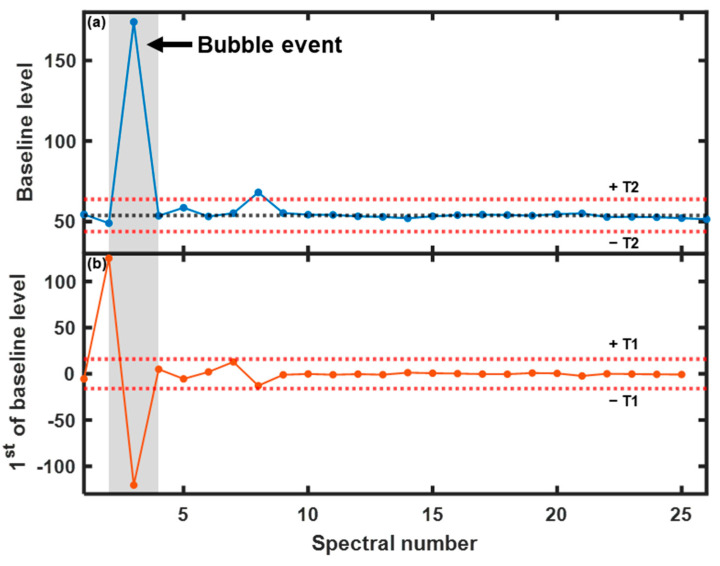
(**a**) Baseline level versus spectral number. The threshold T2 (red dotted line) indicates the range around the determined center (black dotted line). (**b**) First derivative of the baseline level versus spectral number. The threshold T1 (red dotted line) indicates the beginning and end of the bubble event (gray area).

To obtain the beginning and end of a bubble event, the first derivative of the baseline level was determined by calculating the difference between two consecutive values. As the first condition, the position at which the first derivative began to sharply increase was assigned as the beginning of the bubble event, and the position at which the first derivative sharply decreased was assigned as the end of the bubble event, as shown in Figure 5b. The absolute threshold to fulfill this criterion was defined as T1. For the second condition, the baseline level was within a small range around the determined center at the time points when the bubble began and ended, as shown in Figure 5a. The threshold used to identify the range was defined as T2. The signal points that met both conditions indicated the occurrence of bubble events, as shown in Figure 5. More details regarding the conditions are provided in the literature [33]. In this study, the thresholds T1 and T2 were set to 16 and 10, respectively. The sensitivity analysis of T1 and T2 is shown in the Supplementary Materials.

#### 2.4.2. Skewness

The pattern of voidage fluctuation reflects bed fluidity. When the bed fluidity is poor, the dynamic emulsion phase near the wall causes the voidage to fluctuate at a low level, approximately following a normal distribution. When the bed fluidity is good, the probability of large voidage increases because voids occur more frequently due to enhanced bubble behavior. Thus, the statistical values that reflect the distribution of voidage can be used to characterize the bed fluidity. In the study, skewness was used as a fluidity indicator. Because the baseline level of NIR spectra and bed voidage are positively related, the skewness was calculated using the baseline level of NIR spectra.

Skewness represents the degree of asymmetry of a probability density function. If skewness < 0, then most of the data are located on the right and the left tail is longer than the right tail; if skewness > 0, then the peak is toward the left, and the right tail is longer than the left tail. Skewness is defined as the third-order central moment of *bl* divided by the cubic of the standard deviation as follows:

(2) $$S=\frac{\sum_{i=1}^{N}{\left({bl}_{i}-\bar{bl}\right)}^{3}}{(m_{w}-1)\sigma^{3}}$$

where *S* is the skewness of *bl*, *bl_i_* represents the baseline level of the *i*th spectrum in the sliding window, $\bar{bl}$ represents the mean baseline level, σ represents the standard deviation of the baseline level, and *m_w_* is the number of spectra in each sliding window.

### 2.5. Methodology of Fluidity Evaluation

Due to the dynamic characteristics of a fluid bed, the indicator randomly fluctuated with a certain distribution. Thus, it was not reasonable to evaluate the bed fluidity by an arbitrary threshold between the mean values of the indicator for good and poor fluidity. Fluctuations in the indicator could lead to false alarms, including false-positive and false-negative results. Therefore, both the α- and β-errors needed to be controlled in process evaluation [34]. For fluidity detection, the null hypothesis *H*_0_ is that the bed fluidity is good; the alternative hypothesis *H*_1_ is that the bed fluidity is poor. The chance of accepting a case with good fluidity as having poor fluidity was called the α-error, and the chance of accepting a case with poor fluidity as having good fluidity was called the β-error. To establish robust criteria, a threshold was identified based on the above two types of errors from a statistical perspective.

**Figure 6:**
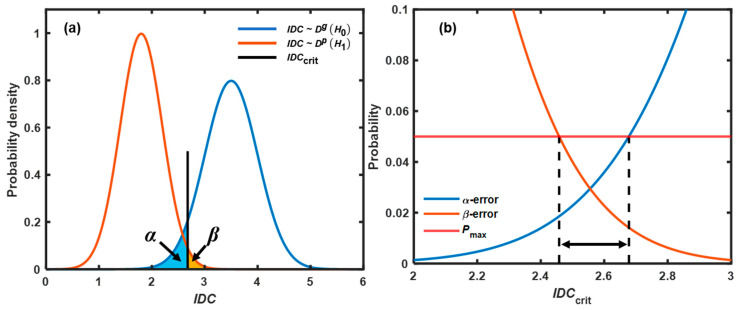
(**a**) Probability density functions (PDFs) of the indicators for *H*_0_ (blue) and *H*_1_ (orange). (**b**) Probabilities of α-errors and β-errors versus *IDC*_crit_. After identifying *P*_max_, shown as the red solid line, *IDC*_crit_ was restricted to a certain range enclosed by the black dashed lines.

First, a large dataset of indicators was prepared to estimate the probability density function (PDF). In this study, 60 data points were obtained from 1560 NIR spectra processed by the sliding window in each calibration experiment. The method proposed by Cai and Hames [35] was used to determine whether the dataset was large enough to make a statistical inference. The results show that the length of the dataset was acceptable. The distributions that the indicator followed under *H*_0_ and *H*_1_ were called $D^{g}$ and $D^{p}$, respectively, as shown in Figure 6a. The reliability of *H*_0_ was confirmed at confidence level 1-α when $IDC\geq {IDC}_{crit}=D_{\alpha }^{g}$. Here, *IDC* was the value of the fluidity indicator calculated from each sliding window, *IDC*_crit_ was the threshold of the fluidity indicator according to the cumulative distribution function (CDF) of $D^{g}$, and α was set to a low level, providing an acceptably low chance of falsely rejecting *H*_0_ (good fluidity). Then, the β-error (chance for falsely rejecting a case of poor fluidity when $IDC\geq {IDC}_{crit}$) was calculated as $\beta =P\left(IDC\geq {IDC}_{crit}\right)$, where $\beta \sim D^{p}$. A typical relationship between α, β, and *IDC*_crit_ is shown in Figure 6b. When *IDC*_crit_ increased, α increased and β decreased. To ensure that the chances of both α-errors and β-errors were less than the threshold of probability (*P*_max_), *IDC*_crit_ was limited to a certain range. In this study, *P*_max_ was set to 15%. The ranges of *IDC*_crit_ for experiments C1–C5 were identified and used for determining a robust threshold, as described in Section 3.3.

## 3. Results and Discussion

### 3.1. Fluidity Characterization by Visual Observation

**Figure 7:**
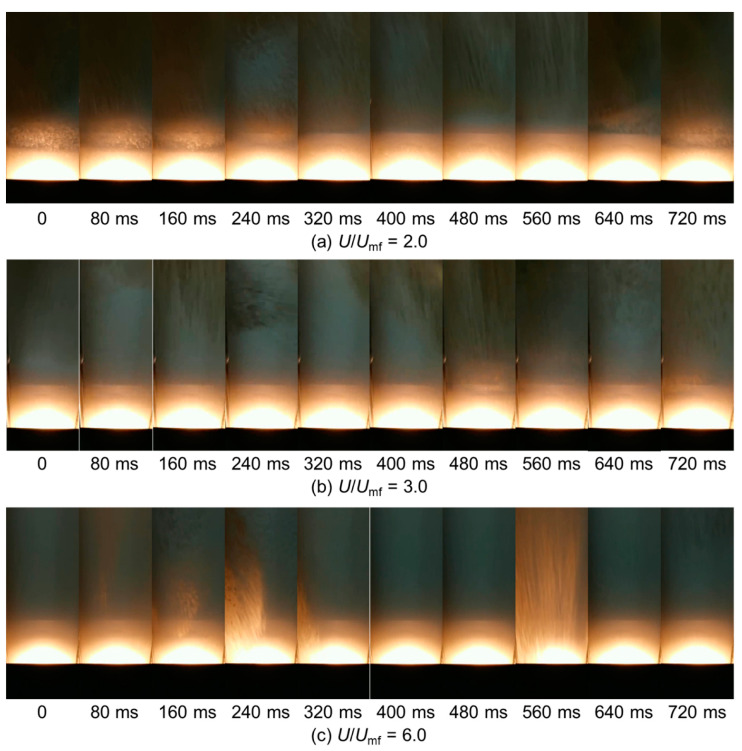
Images of the bed flow pattern evolving over time at three superficial gas velocities: (**a**) *U*/*U*_mf_ = 2.0, (**b**) *U*/*U*_mf_ = 3.0, and (**c**) *U*/*U*_mf_ = 6.0. *d*_p_ = 630 μm; *H*_b_ = 12 cm.

The bed fluidity was identified as poor, medium, or good based on visual observation of the movement of bed material through the sight window. With increasing superficial gas velocity, the bed flow pattern moved through three stages: stepwise, free-flowing, and vigorous, corresponding to poor, medium, and good fluidity, respectively. When the gas velocity was low, the downward movement of bed material at the wall was stepwise. As shown in Figure 7a, the particle movement was inactive. Bubbles rarely passed near the wall and finally erupted from the center of the bed surface. As the superficial gas velocity was further increased, the downward particle circulation changed from a stepwise pattern to a more continuous, free-flowing pattern. As shown in Figure 7b, the particle circulation velocity was enhanced, and the instant of the packed bed was no longer observed. A similar conversion was also observed in a fluidized bed drying experiment [36]. If the superficial gas velocity continued to increase, the bubble behavior became more intense, and bubbles began to appear frequently at the wall, as shown in Figure 7c. Videos for three different flow patterns are presented in the Supplementary Materials. Due to the enhanced interaction between the emulsion phase and bubble phase, the regular circulation of the particles was disrupted. The degree of irregularity increased with increasing gas velocity. The flow pattern under this condition was defined as vigorous. The summary of bed fluidity with different *U*/*U*_mf_ values in each calibration experiment is provided in Table 3.

### 3.2. Effect of Operating Conditions on the Fluidity Indicators

#### 3.2.1. Effect of Static Bed Height

**Figure 8:**
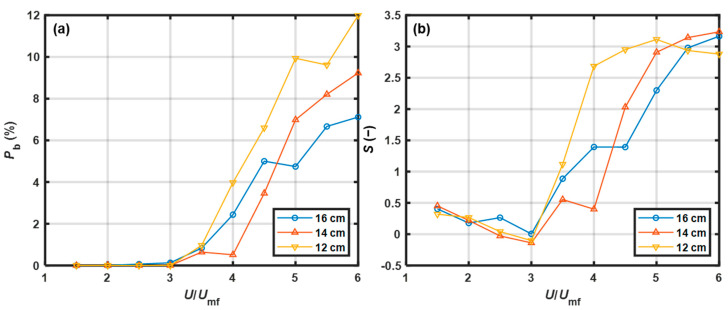
Effect of the static bed height on changes in (**a**) bubble proportion and (**b**) skewness with *U*/*U*_mf_ (*d*_p_ = 805 μm).

The effect of the static bed height on the bubble proportion as a function of *U*/*U*_mf_ is shown in Figure 8a. When *U*/*U*_mf_ < 3.0, *P*_b_ was approximately zero, indicating that bubbles rarely appeared at the walls. According to the visual observation results listed in Table 3, the bed showed a stepwise pattern or changed to the free-flowing pattern, and bubble behavior was inactive over a range of low gas velocities. When *U*/*U*_mf_ was beyond the range, the curves entered a growth state. This potentially occurred because the bubble number increased with superficial gas velocity; thus, the chance of detecting bubbles near the wall increased. At high gas velocities, *P*_b_ decreased with increasing static bed height. Jang et al. [37] stated that the fraction of large bubbles decreased with increasing static bed height. Because large bubbles were more likely to be detected when the static bed height was lower, this theory was consistent with our experimental results.

The effect of the static bed height on skewness as a function of *U*/*U*_mf_ is shown in Figure 8b. When *U*/*U*_mf_ < 3.0, the skewness was negatively correlated with superficial gas velocity. The skewness changed from positive to negative for the bed with a *H*_b_ of 12 cm and 14 cm or to nearly zero for the bed with a *H*_b_ of 16 cm. As the gas velocity was low, the bed showed a stepwise pattern, most of the data originated from a packed bed with small void spaces, and the right tail was composed of extreme values originating from a free-flowing bed with large void spaces. With increasing gas velocity, the proportion of the time during which the bed was free-flowing increased, such that the peak of the baseline level gradually developed a bias toward the right. When *U*/*U*_mf_ > 3.0, the skewness sharply increased to approximately 3 with increasing superficial gas velocity, which indicated that the data for the baseline level were skewed toward the right. As the gas velocity increased, the probability of bubble events at the wall increased. The right tail of the PDF became longer owing to an increase in the extremely high voidage values that resulted from bubble events, and this corresponded to greater positive skewness. At high gas velocities, the curves entered a relatively stable state. Additionally, a smaller static bed height correlated with a faster-developed stable state.

#### 3.2.2. Effect of Particle Size

**Figure 9:**
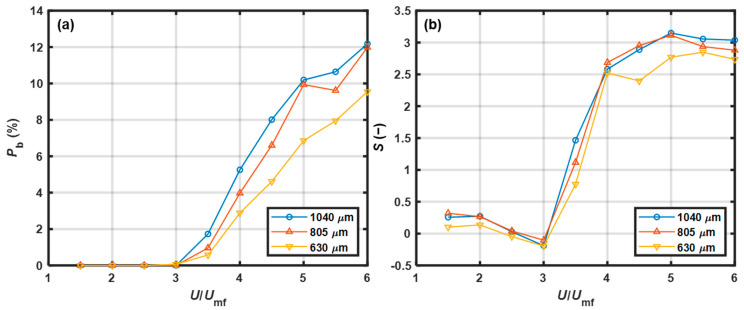
Effect of particle size on the change in (**a**) bubble proportion and (**b**) skewness with *U*/*U*_mf_ (*H*_b_ = 12 cm).

The effect of the particle size on the bubble proportion as a function of *U*/*U*_mf_ is shown in Figure 9a. When *U*/*U*_mf_ > 3.0, *P*_b_ was positively correlated with particle size. At high gas velocities, the difference between the 630 μm and 850 μm PP particles was larger than that between the 850 μm and 1040 μm PP particles. As the particle size increased, the ability of the air gas to fluidize particles decreased, and the particles became densely distributed in the lower section of the fluidized bed [38]. Hence, the solid volume fraction decreased in the upper section of the bed where the NIR probe was positioned, which led to a larger *P*_b_.

The effect of the particle size on the skewness as a function of *U*/*U*_mf_ is shown in Figure 9b. When *U*/*U*_mf_ < 3.0, the skewness was below 0.5 and slowly decreased with increasing gas velocity. Then, the skewness sharply increased to approximately 2.5 over the range of *U*/*U*_mf_ from 3.0 to 4.0. As the gas velocity continued to increase, the curves slowly increased and then entered the stable state. The skewness of the larger particles was larger when *U*/*U*_mf_ was above 5.0. The solid volume fraction in the upper section of the bed decreased with increasing particle size. Thus, in the bed with larger particles, the baseline levels of the NIR spectra were greater due to the transmission effect caused by the reduction in solid volume fraction. This resulted in a longer tail on the right side of the PDF, which caused greater positive skewness.

### 3.3. Establishment and Validation of the Fluidity Evaluation Method

#### 3.3.1. Bubble Proportion

As shown in Figure 8a and Figure 9a, *P*_b_ increased with superficial gas velocity, indicating an improvement in bed fluidity. When the bed fluidity was poor, *P*_b_ was nearly zero. However, when the bed fluidity was good, *P*_b_ increased to a high level. The fluidity evaluation method based on the bubble proportion was developed as follows: if *P*_b_ was zero, then the bed fluidity was evaluated as poor; if *P*_b_ was not less than 3.85%, corresponding to at least one spectrum with a high baseline level within the sliding window, the bed fluidity was evaluated as good. Thus, the threshold was set to 3.85%.

**Figure 10:**
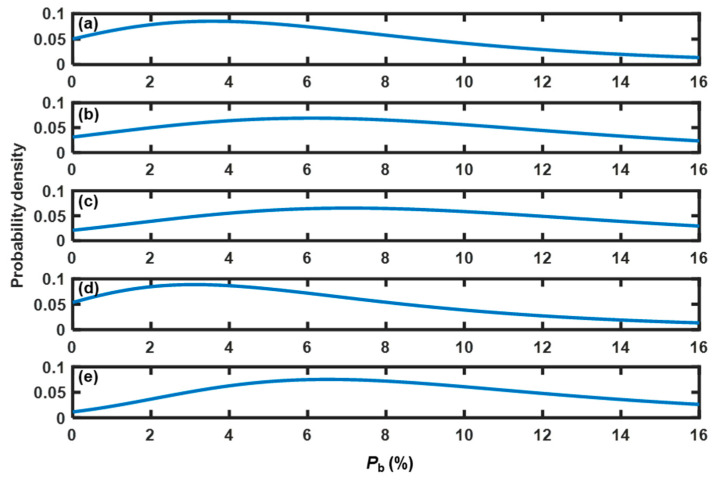
PDFs of *P*_b_ under good fluidity in the calibration experiments: (**a**) C1 (*d*_p_ = 805 μm, *H*_b_ = 16 cm, *U*/*U*_mf_ = 5.5), (**b**) C2 (*d*_p_ = 805 μm, *H*_b_ = 14 cm, *U*/*U*_mf_ = 5.5), (**c**) C3 (*d*_p_ = 805 μm, *H*_b_ = 12 cm, *U*/*U*_mf_ = 5.0), (**d**) C4 (*d*_p_ = 630 μm, *H*_b_ = 12 cm, *U*/*U*_mf_ = 5.0), and (**e**) C5 (*d*_p_ = 1040 μm, *H*_b_ = 12 cm, *U*/*U*_mf_ = 5.0).

According to the method described in Section 2.5, both α-errors and β-errors were considered to evaluate the threshold. Because *P*_b_ for poor fluidity was constant (zero), β-error was avoided when setting the threshold of *P*_b_ as 3.85%. To calculate the chance of α-error, the first step was to estimate the PDF that *P*_b_ followed in each calibration experiment. The fitting results indicate that *P*_b_ followed a general extreme value (GEV) distribution, and the PDFs are shown in Figure 10. Then, the chance of α-error was equal to the cumulative distribution probability at a *P*_b_ of 3.85%. As shown in Table 4, except for experiment C5, all chances of α-error were above the probability limit of 15%. Therefore, the indicator *P*_b_ was unsatisfactory for the detection of bed fluidity.

#### 3.3.2. Skewness

**Figure 11:**
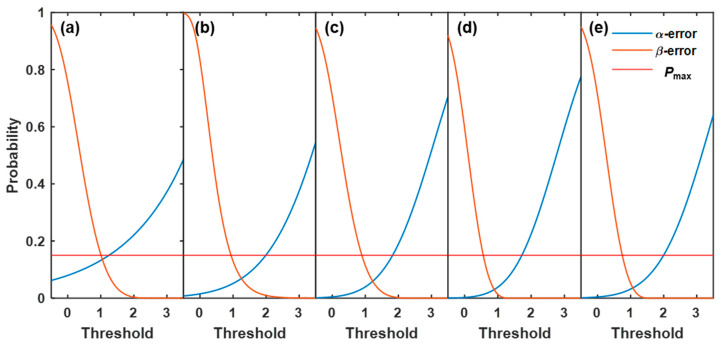
Relationships between the threshold value and the probabilities of α-errors and β-errors in the calibration experiments: (**a**) C1 (*d*_p_ = 805 μm, *H*_b_ = 16 cm), (**b**) C2 (*d*_p_ = 805 μm, *H*_b_ = 14 cm), (**c**) C3 (*d*_p_ = 805 μm, *H*_b_ = 12 cm), (**d**) C4 (*d*_p_ = 630 μm, *H*_b_ = 12 cm), and (**e**) C5 (*d*_p_ = 1040 μm, *H*_b_ = 12 cm).

As shown in Figure 8b and Figure 9b, the skewness values were higher for good fluidity than those for poor fluidity. Therefore, the fluidity evaluation method based on skewness was developed as follows: if the skewness was above the threshold, then the bed fluidity was evaluated as good. Otherwise, the bed fluidity was poor. To obtain the relationships between the threshold value and the chances of α-errors and β-errors, the PDFs of skewness at good and poor fluidity were initially determined. The datasets of skewness used to estimate the PDFs at good fluidity in experiments C1–C5 were obtained at *U*/*U*_mf_ = 5.5, 5.5, 5.0, 5.0, and 5.0, respectively. These superficial gas velocities were critical velocities at which the fluidity changed from medium to good. The datasets of skewness with *U*/*U*_mf_ = 1.5 were selected to estimate the PDFs at poor fluidity. The fitting results indicate that all the datasets of skewness followed a GEV distribution at good and poor fluidity. The probabilities of α-errors and β-errors as functions of the threshold in each calibration experiment are shown in Figure 11. To obtain a robust threshold, the ranges of acceptable thresholds over which both probabilities of α- and β-errors were less than 15% in experiments C1–C5 were determined, as listed in Table 5. In the study, the skewness threshold was set to 1.20. There was no error with a probability greater than 15% as listed in Table 4, indicating that the fluidity detection method based on skewness with a threshold of 1.20 was effective under varied conditions.

#### 3.3.3. Method Validation

**Figure 12:**
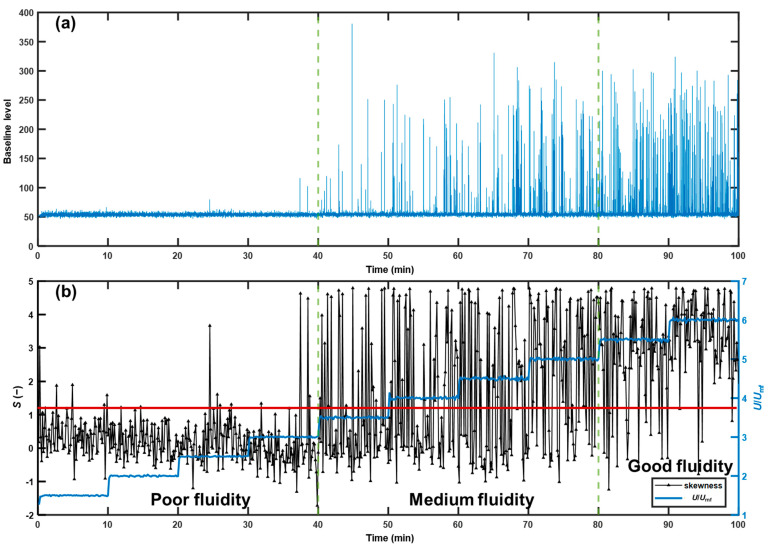
Profiles of (**a**) baseline level and (**b**) skewness and superficial gas velocity in experiment V1. The boundaries between different bed fluidities are indicated by green dashed lines. The skewness threshold is 1.20, as indicated by the red solid line.

Several experiments were conducted to validate the established fluidity detection method based on skewness. A series of experiments were performed under varied conditions for robustness evaluation, as shown in Table 2. Experiment V1 was used as an example. Figure 12a illustrates a representative baseline level curve when the bed fluidity changed from poor to good. The frequencies of extreme values of baseline level increased with increasing superficial gas velocity, indicating that the voidage fluctuated more vigorously for good fluidity than poor fluidity, which was consistent with visual observations. As shown in Figure 12b, when the bed showed poor fluidity, most points of skewness were below the threshold of 1.20. When the bed showed medium fluidity, the skewness fluctuated in a wider range. As the bed reached good fluidity, most of the points were concentrated above the threshold of 1.20. These results validate that the skewness of the baseline level could be used to evaluate bed fluidity. The chance of β-error was calculated from the ratio of the point number of skewness greater than 1.20 to the total point number in the *U/U*_mf_ range from 1.5 to 3.0, during which the bed was judged under poor fluidity by visual observation; while the chance of α-error was calculated from the ratio of the point number of skewness less than 1.20 to the total point number in the *U/U*_mf_ range from 5.5 to 6.0, during which the bed fluidity was assessed as good by visual observation. The summary of α-errors and β-errors in the validation experiments is provided in Table 6. The probabilities of the α-errors and β-errors were both less than 15% as expected. Therefore, the fluidity detection method based on skewness had suitable robustness for application in fluidization processes with variable static bed heights and particle sizes.

**Figure 13:**
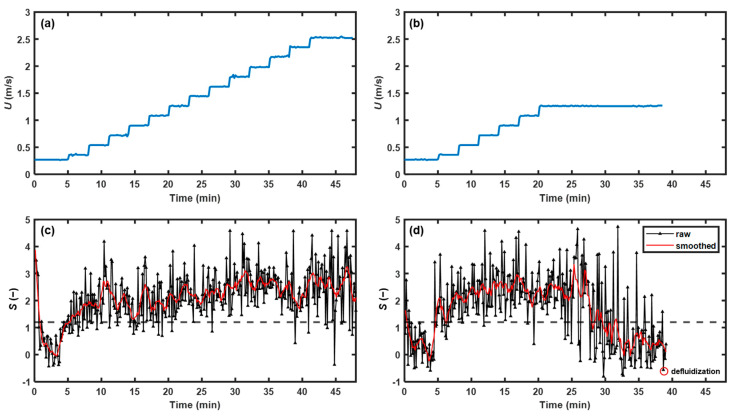
Profiles of the superficial gas velocity in (**a**) normal fluidized bed granulation process and (**b**) abnormal fluidized bed granulation; skewness in (**c**) normal fluidized bed granulation process and (**d**) abnormal fluidized bed granulation. The skewness threshold is 1.20, as indicated by the black solid line.

To demonstrate its applicability, the NIR method was applied to monitor bed fluidity in real time for the normal and abnormal fluidized bed granulation processes. The superficial gas velocity increased stepwise in normal fluidized bed granulation process, as shown in Figure 13a. The bed fluidity was maintained throughout the spraying phase. As shown in Figure 13c, most points of skewness were above the threshold of 1.20, indicating good bed fluidity, which was consistent with visual observation. Notably, the skewness was less than 1.20 in the first 5 min because the window was covered with cohesive powder, causing misleading spectral information. Once the powder was flushed away, the indicator rapidly increased to a normal level. In the abnormal fluidized bed granulation process, the superficial gas velocity increased stepwise in the first 20 min and then remained constant, as shown in Figure 13b. After 20 min, the drag force exerted on the particles stopped increasing, while the particle size and moisture continued to grow, leading to an increase in gravity and liquid bridge force. Thus, the force equilibrium of the particle was disrupted, and then the bed fluidity gradually deteriorated, resulting in defluidization at 38 min. As shown in Figure 13d, most indicator points were above the threshold before 28 min, and then the skewness showed a downward trend, indicating the start of fluidity deterioration. After approximately 33 min, most indicator points were concentrated under the threshold until bed collapse. Therefore, the NIR method was feasible in monitoring bed fluidity and warning of defluidization in practice.

## 4. Conclusions

The baseline drift of diffuse reflectance NIR spectroscopy can be used to characterize the fluidity of gas–solid fluidized beds. In this study, diffuse reflectance NIR spectra were acquired by using an NIR probe installed next to the sight window. The baseline level of the diffuse reflectance NIR spectra, which was defined as the sum of the values of log_10_(1/*R*) at different wavelengths, was used to quantify the voidage near the wall of the fluidized bed. Two indicators were extracted from the baseline fluctuation profiles, called the bubble proportion and skewness, and used to characterize the bed fluidity. Both indicators were sensitive to changes in bed fluidity under varied conditions, including the static bed height and particle size. Considering both α- and β-errors, a robust skewness threshold was identified to distinguish poor and good fluidity under varied conditions. Then, the fluidity detection method based on skewness was established as follows: if the skewness was larger than 1.20, the bed fluidity was evaluated as good. Otherwise, the bed fluidity was poor. Both the robustness and applicability of the NIR method were validated. The probability of a false alarm was shown to be less than 15% under varied material conditions. With the developed method, diffuse reflectance NIR spectroscopy was successfully used for fluidity detection in a laboratory-scale fluidized bed granulation process. Based on the results from our study, diffuse reflectance NIR spectroscopy is a cost-effective PAT tool, using the same instrument for both the granule attribute quantification and fluidization quality detection.

## Figures and Tables

**Table 1:** Conditions of calibration experiments for method development.

Experiment Number	*d*_p_ (μm)	*H*_b_ (cm)	*U*_mf_ (m/s)	*U*/*U*_mf_
C1	805	16	0.33	1.5, 2.0, 2.5, 3.0, 3.5 4.0, 4.5, 5.0, 5.5, 6.0
C2	805	14	0.32	
C3	805	12	0.30	
C4	630	12	0.26	
C5	1040	12	0.33	

**Table 2:** Conditions of validation experiments for robustness evaluation.

Experiment Number	*d*_p_ (μm)	*H*_b_ (cm)	*U*/*U*_mf_
V1	805	16	1.5–6.0
V2	805	14	1.5–6.0
V3	805	12	1.5–6.0
V4	630	12	1.5–6.0
V5	1040	12	1.5–6.0

**Table 3:** Summary of bed fluidity characterized by visual observation.

*U*/*U*_mf_	Experiment C1		Experiment C2		Experiment C3		Experiment C4		Experiment C5	
	Flow Pattern	Fluidity	Flow Pattern	Fluidity	Flow Pattern	Fluidity	Flow Pattern	Fluidity	Flow Pattern	Fluidity
1.5	P1 ^1^	P ^2^	P1	P	P1	P	P1	P	P1	P
2.0	P1	P	P1	P	P1	P	P1	P	P1	P
2.5	P1	P	P1	P	P1	P	P1	P	P1	P
3.0	P1	P	P2	M	P2	M	P2	M	P2	M
3.5	P2	M	P2	M	P2	M	P2	M	P2	M
4.0	P2	M	P2	M	P2	M	P2	M	P2	M
4.5	P2	M	P2	M	P2	M	P2	M	P2	M
5.0	P2	M	P2	M	P3	G	P3	G	P3	G
5.5	P3	G	P3	G	P3	G	P3	G	P3	G
6.0	P3	G	P3	G	P3	G	P3	G	P3	G

^1^ P1, P2, and P3 refer to stepwise, free-flowing, and vigorous patterns, respectively. ^2^ P, M, and G refer to poor, medium, and good fluidity, respectively.

**Table 4:** Summary of the α-errors and β-errors in the calibration experiments.

Indicator	Threshold Value	Error Type	C1	C2	C3	C4	C5
*P*_b_	3.85	α	36.7%	25.5%	18.3%	37.9%	14.9%
Skewness	1.20	α	14.7%	6.5%	5.7%	5.9%	4.5%
		β	9.0%	7.9%	6.4%	0.1%	1.5%

**Table 5:** The ranges of acceptable thresholds in calibration experiments.

Skewness	C1	C2	C3	C4	C5
range	1.01–1.24	0.94–1.98	0.90–1.82	0.55–1.71	0.75–1.99

**Table 6:** Summary of the α-errors and β-errors in the validation experiments for robustness evaluation.

Indicator	Threshold Value	Error Type	V1	V2	V3	V4	V5
Skewness	1.20	α	12.7%	9.3%	3.1%	8.4%	4.0%
		β	5.3%	3.6%	3.1%	1.8%	3.1%

## Data Availability

Not applicable.

## Supplementary Materials

The following supporting information can be downloaded at: https://www.mdpi.com/article/10.3390/pharmaceutics15092246/s1, Figure S1: (a) The influence of threshold T1 on the detection accuracy of bubble events (T2 = 10). (b) The influence of threshold T2 on the detection accuracy of bubble events (T1 = 16). The signal points assigned to bubble events are marked by red circles. The determined baseline and threshold T2 are indicated by the black dotted line and red dotted line, respectively; Figure S2: The bubble proportion Pb versus threshold for each experiment. (a) Sensitivity toward threshold T1 (T2 = 10). (b) Sensitivity toward threshold T2 (T1 = 16); Video S1: Free-flowing flow pattern; Video S2: Stepwise flow pattern; Video S3: Vigorous flow pattern.
