# Supplementary material for: In-Line Detection of Bed Fluidity in Gas–Solid Fluidized Beds Using Near-Infrared Spectroscopy
Source: Pharmaceutics. 2023 Aug 30;15(9):2246. doi: 10.3390/pharmaceutics15092246 (PMC10537685; doi:10.3390/pharmaceutics15092246)
Supplement: Supplementary file 1 [file pharmaceutics-15-02246-s001.zip › Supplementary Materials.pdf]

Supplementary Data for

In-Line Detection of Bed Fluidity in Gas–Solid Fluidized Beds Using Near-Infrared Spectroscopy

Hao Fu, Kaixuan Teng, Jie Zhao, Sheng Zhang and Haibin Qu

Sensitivity analysis of the thresholds adopted in the bubble event detection algorithm

After quantifying the fluctuation degree by the first-order derivative of baseline level, the threshold T1 was used to identify whether the NIR spectra originated from the emulsion phase or the bubble phase. If T1 was too low, the fluctuations of baseline caused by the emulsion phase were misinterpreted as originating from conversion between the emulsion phase and the bubble phase. The 48-56 s signal (26 NIR spectra) of experiment C3 with $U/U_{mf} = 6.0$ was taken as an example. As shown in Figure S1(a), if T1 was set as 4 or 8, then point 12 was misjudged as the signal from the bubble phase. When T1 was set equal to or larger than 16, then the signals from the emulsion and bubble phase were identified accurately. However, if T1 was set too high, then small bubble events were missed. For T2, the threshold also influenced the detection results of the beginning and end of the bubble events. If T2 was too low, then the beginning or end of the bubble events was not detected. As shown in Figure S1(b), when T2 was set to 8, point 24, which was assigned to a bubble event, was not detected. However, if T2 was too high, then the bubble events were determined

to start late or end early, which led to a small P_b.

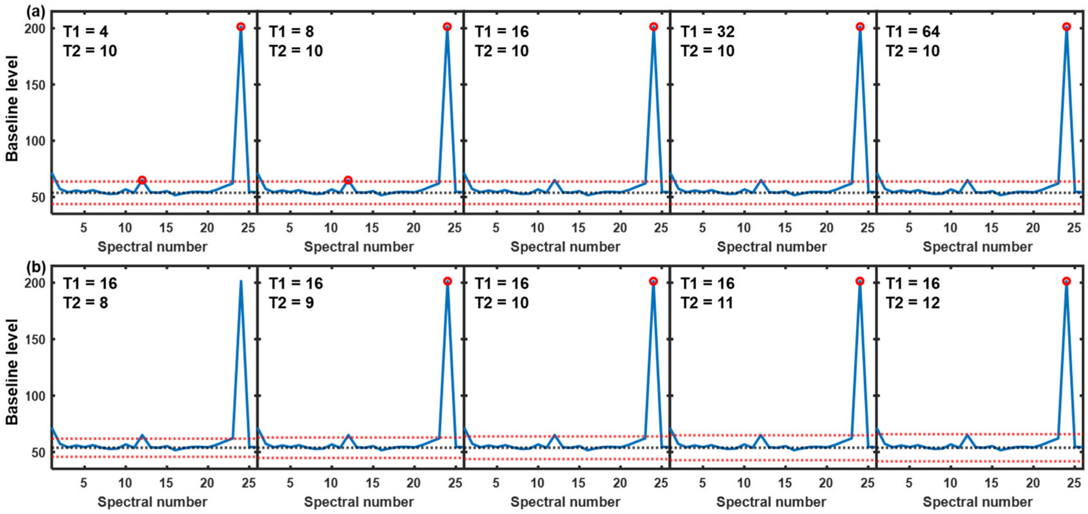

Figure S1. (a) The influence of threshold T1 on the detection accuracy of bubble events (T2 = 10). (b) The influence of threshold T2 on the detection accuracy of bubble events (T1 = 16). The signal points assigned to bubble events are marked by red circles. The determined baseline and threshold T2 are indicated by the black dotted line and red dotted line, respectively.

The first 26 NIR spectra collected during 0-8 s of each calibration experiment with U/U_{mf} = 6.0 were selected for a sensitivity analysis. Figure S2(a) shows the influence of threshold T1 applied to the first derivative to determine the beginning and end of a bubble. The value of the threshold T1 varied in a range between 4 and 64. The P_b gradually became stable with the increase in the threshold T1 from 4 to 32. However, when was T2 increased to 64, small bubble

events were missed, leading to an abnormal P_b, which occurred in experiments C4 and C5. Thus, the value of threshold T1 was set to 16 in this study. Figure S2(b) shows the results of the sensitivity analysis of threshold T2. The value of threshold T2 increased from 8 to 12. However, no significant effect of the threshold value on the P_b was detected, and thus a threshold value of 10 was selected in this study.

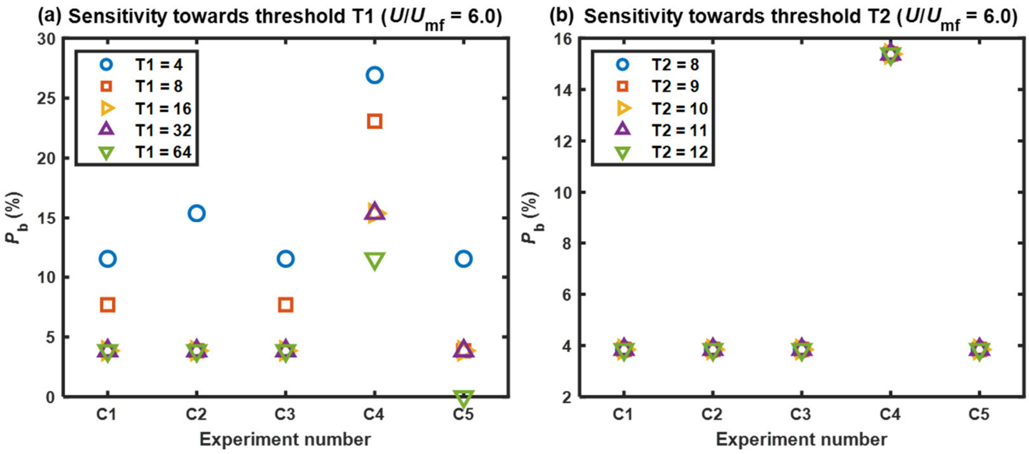

Figure S2. The bubble proportion P_b versus threshold for each experiment. (a) Sensitivity toward threshold T1 (T2=10). (b) Sensitivity toward threshold T2 (T1=16).
